# Comparative analysis of biofilm characterization of probiotic *Escherichia coli*

**DOI:** 10.3389/fmicb.2024.1365562

**Published:** 2024-03-15

**Authors:** Huiping Liu, Jingfang Ma, Pan Yang, Feng Geng, Xueling Li, Junhong Lü, Yadi Wang

**Affiliations:** ^1^College of Pharmacy, Binzhou Medical University, Yantai, China; ^2^Yantai Affiliated Hospital of Binzhou Medical University, Yantai, China; ^3^Shanghai University of Medicine & Health Sciences, Shanghai, China; ^4^Jinan Microecological Biomedicine Shandong Laboratory, Jinan, China

**Keywords:** biofilms, infrared spectroscopy, *Escherichia coli*, probiotics, spatial heterogeneity

## Abstract

Biofilms are thought to play a vital role in the beneficial effects of probiotic bacteria. However, the structure and function of probiotic biofilms are poorly understood. In this work, biofilms of *Escherichia coli* (*E. coli*) Nissle 1917 were investigated and compared with those of pathogenic and opportunistic strains (*E. coli* MG1655, O157:H7) using crystal violet assay, confocal laser scanning microscopy, scanning electron microscopy and FTIR microspectroscopy. The study revealed significant differences in the morphological structure, chemical composition, and spatial heterogeneity of the biofilm formed by the probiotic *E. coli* strain. In particular, the probiotic biofilm can secrete unique phospholipid components into the extracellular matrix. These findings provide new information on the morphology, architecture and chemical heterogeneity of probiotic biofilms. This information may help us to understand the beneficial effects of probiotics for various applications.

## 1 Introduction

Probiotics, owing to their numerous benefits to host health, have attracted extensive attention in alternative therapy for various diseases (Lynch et al., 2022). *E. coli* Nissle 1917, for instance, has been used as a probiotic in clinical treatments for many decades, successfully alleviating intestinal disorders such as ulcerative colitis and inflammatory bowel disease (Michael et al., 2021; Lynch et al., 2022; Zhao R. et al., 2022; Zhao Z. et al., 2022). Numerous studies have demonstrated that biofilm formation by probiotic bacteria is a beneficial property through various mechanisms (Fokt et al., 2022; Ramadevi et al., 2023). When the probiotic bacteria colonize on the mucosal surface of the host, they form biofilm to promote colonization and increase their longer permanence while impeding the infection and colonization of other intestinal microorganisms including opportunistic and pathogenic bacteria (Bartram et al., 2023). The probiotics cloud not only stimulate the host’s immune response but also promote the secretion of antimicrobials (Lynch et al., 2022). The ability of different bacterial species to form biofilms and the harmful effects of biofilms associated with pathogenic bacteria have been extensive studied (Hancock et al., 2010a). A lack of detailed characterization and comparison of biofilms hinders our understanding of the underlying mechanisms of probiotic biofilms. Further research in this area is important to fully understand the potential beneficial properties of probiotic biofilms.

*Escherichia coli* is a non-sporulating, rod-shaped, Gram-negative bacterium commonly used as a model organism in bioengineering and industrial microbiology. While some pathogenic strains of *E. coli* can cause severe disease (Wasinski, 2019), it is important to note that the majority of *E. coli* in the normal gut microbiota are harmless. In fact, the presence of probiotic *E. coli* in the intestine can actively benefit the host by preventing the colonization of pathogenic bacteria. Bacteria commonly exist in biofilms. In nature, bacteria often exist in biofilms, which provide them with protection and stability in changing environments (Sharma et al., 2016). This makes them more resistant to antimicrobials than planktonic cells. Biofilms are complex communities of sessile bacteria that are characterized by the presence of a matrix surrounding the bacterial cells. The matrix contains extracellular polysaccharides, proteins, and extracellular DNA (Sharma et al., 2016; Rossi et al., 2018; Al-Kafaween et al., 2019). However, the differences between *E. coli* strains in biofilm formation and chemical structure have not yet been explored (Stewart and Franklin, 2008; Hancock et al., 2010b; Beitelshees et al., 2018).

Here we used various techniques, including crystal violet staining, confocal laser scanning microscopy, scanning electron microscopy and FTIR microspectroscopy, to investigate the *E. coli* biofilm formed by probiotic strains. We also compared their biofilm differences in morphology, structural composition, and spatial heterogeneity with pathogenic and opportunistic *E. coli* strains under different culture conditions. The results obtained would be help to provide mechanistic insight into the probiotic properties of biofilms for potential application.

## 2 Materials and methods

### 2.1 Biofilm culture

Three strains of *E. coli* namely MG1655, Nissle 1917 and O157:H7 were used in this study. MG1655 was obtained from the Institute of Engineering and Biotechnology, O157:H7 (CICC 21530) was obtained from China Center of Industrial Culture Collection, Nissle 1917 (DM6601) was obtained from German Collection of Microorganisms and Cell Cultures.

Three bacterial strains were inoculated into Luria-Bertani (LB) broth medium and let to grow at 37°C, 220 rpm till logarithmic growth period, respectively. OD600 of the bacterial suspension was measured and adjusted to Abs = 1 (approximately 1 × 10^9^ CFU/mL) using fresh LB broth, and then it was further diluted 100 times to OD600 = 0.01. Then they were transferred into a 24-well cell culture plate (Corning, CLS3524) respectively. Then they were incubated at 25°C for 12, 24, 36, 48, or 60 h. After that, supernatant containing planktonic cells were removed and the wells were gently rinsed thrice with pure water.

### 2.2 Crystal violet (CV) assay

Biofilms were stained with 0.1% (V/V) crystal violet for 20 min, rinsed three times with pure water, and the wells were dried completely. 95% (V/V) ethanol was added to each well and kept for 15 min to resuspend the stained biofilms completely. The resulting absorbance at 595 nm was measured using a spectrophotometer.

### 2.3 Confocal laser scanning microscopy (CLSM) characterization

Bacterial cells with an initial concentration of OD600 = 0.01 in LB broth medium were transferred into glass-bottomed dishes (Cellvis, D29-14-1-N) and incubated at 25°C for 24 or 48 h to form biofilms, respectively. Subsequently, supernatant was removed and the biofilms attached on the dish were rinsed gently three times in PBS. The biofilms were then stained with SYTO 9 (Invitrogen, L13152) according to the manufacturer’s instructions, briefly, 3 μM SYTO 9 stain was added to the biofilms and incubate at room temperature in the dark for 15 min. Then it was examined using confocal laser scanning microscopy (ZEISS LSM 880) with an excitation of 488 nm. Three-dimensional images of the biofilms were generated through Z-stack imaging, utilized the Zen Black software (Zeiss, Oberkochen, Germany). Image J was subsequently used to calculate the integrated density.

### 2.4 Scanning electron microscopy (SEM) characterization

Bacterial cells with an initial concentration of OD600 = 0.01 in LB broth medium were transferred into a 24-well cell culture plate respectively, with each well containing a piece of sterile cover slip. Then the plates were incubated in a 25°C incubator for 24 or 48 h. Biofilms would grow and attach on cover slips. Prior to observation, the biofilms were fixed using a 2.5% (V/V) glutaraldehyde solution for 4 h, followed by rinsing with PBS three times. Gradient dehydration was performed using ethanal concentrations (V/V) of 30, 50, 70, 80, 95, and 100% for ten minutes each. After CO_2_ critical point drying and gold sputter coating, the samples were observed using a scanning electron microscope (Zeiss, Germany, EVO LS15).

### 2.5 FTIR microspectroscopy experiment

Bacterial cells with an initial concentration of OD600 = 0.01 in LB broth medium were transferred into a 24-well cell culture plate respectively, with each well containing a piece of sterile CaF_2_ window. Then the plates were incubated in a 25°C incubator for 48 h. Subsequently, the CaF_2_ windows were rinsed three times with deionized water to eliminate any planktonic microorganisms. The windows were then immersed in a 4% paraformaldehyde solution for 30 min, followed by another wash with deionized water and left to dry at room temperature. Finally, FTIR microspectroscopy investigations were carried out at the beamline BL01B and BL06B of Shanghai Synchrotron Radiation Facility (SSRF). The aperture was set to 30 × 30 μm with a step size of 15 × 15 μm. The spectral range was 4,000–650 cm^−1^ with a spectral resolution of 4 cm^−1^, and each spectrum comprised 32 co-added scans.

### 2.6 Data analysis

Following IR mapping images, 9-point smoothing, baseline correction and normalization on CytoSpec were carried out. PCA (principal component analysis) and heterogeneity analysis were subsequently executed on Matlab R2021b, as detailed in our previous publications (Baker et al., 2014; Wang et al., 2023a,b).

Hypothesis testing was conducted to characterize the significance of differences between groups by GraphPad Prism7. Then, the IR peak positions of each biofilm were statistically analyzed and compared by two-sample *t* test.

## 3 Results and discussion

### 3.1 Biofilm mass estimation

**Figure 1:**
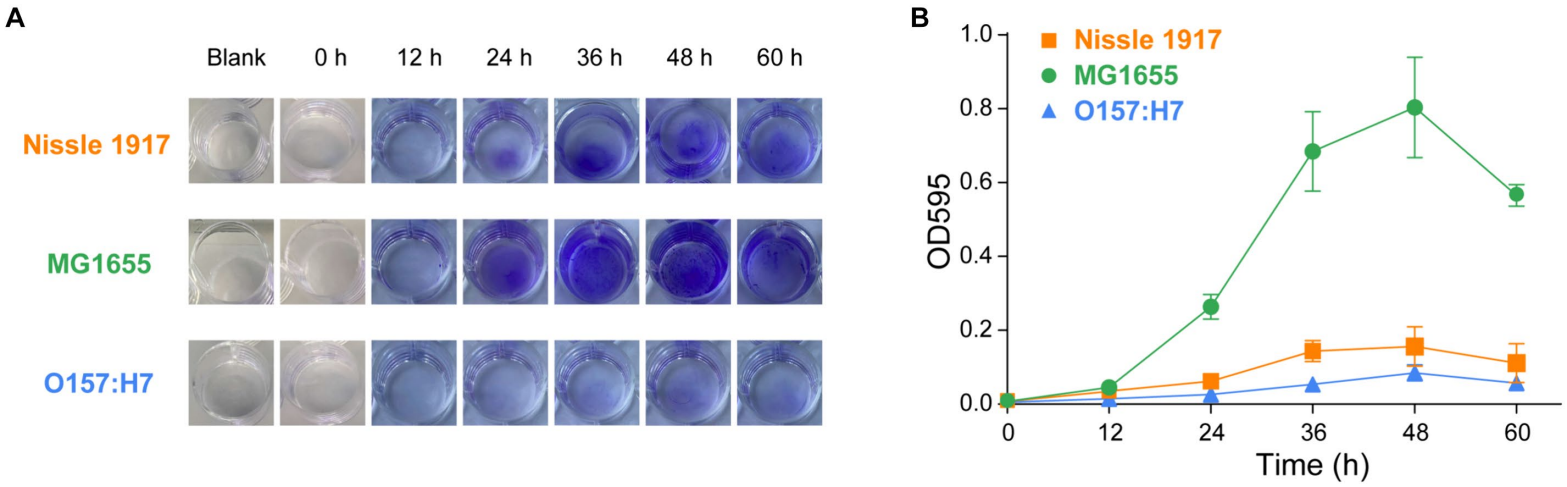
Comparison of the biofilm formation ability of probiotic *E. coli* strain with opportunistic and pathogenic strains, accessed using crystal violet (CV) staining. The biofilms of probiotic *E. coli* strain Nissle 1917, opportunistic pathogenic strain MG1655 and pathogenic strain O157:H7 biofilms were allowed to grow for 0, 12, 24, 36, 48, and 60 h. Blank indicated the wells containing only culture media and incubated for 60 h. The mass of biofilms was then measured using CV staining. Images of the biofilms can be seen in **(A)** and the corresponding values of CV absorbance at 595 nm are presented in **(B)**.

The ability of the probiotic *E. coli* Nissle 1917 to form biofilms is compared with the opportunistic strain MG1655 and the pathogenic strain O157:H7. Crystal violet (CV) is a membrane-permeable basic trianiline dye that can be used to quantitatively assess biofilm biomass (Lianou and Koutsoumanis, 2012; Wilson et al., 2017). Three *E. coli* strains were inoculated into 24-well plates and incubated at 25°C each (Mathlouthi et al., 2018). After staining with CV, the formed biofilms were quantitatively analyzed by measuring the optical density at 595 nm. As shown in Figures 1A,B, the biofilm formation ability of MG1655 was the strongest, followed by Nissle1917, while O157:H7 was the weakest. The biomasses of biofilm formation of the strains were low at 12 h, then increased significantly from 24 h and increased along with time to reach the maximum value at 48 h, after which it started to decrease. This meant that the three *E. coli* biofilms started to enter the attachment stage at about 12 h, then enter the colonization and development stage from 24 h to reach the maturity stage at about 48 h, after which they started to disperse and colonize elsewhere, so the density of the organisms may start to decrease (Nag and Lahiri, 2021). Apart from this, when the biofilms are fully mature, the lack of sufficient space and nutrition will also lead to the death of microbial cells (Tahmourespour, 2012). The biofilm mass of the three strains started to differ significantly at 24 h, at which time the amount of biofilm formed in Nissle 1917 is 4.2 times less than MG1655, and 2.4 times more than O157:H7. At both 48 h and 60 h, the amount of biofilm formed by Nissle 1917 is approximately 5 times less than MG1655, and 2 times more than O157:H7. In conclusion, the biofilms of the three *E. coli* strains all enter the colonization and development stage from 24 h and enter the maturity stage at around 48 h, with MG1655 forming more the amount of biofilm mass, followed by Nissle 1917 and O157:H7. Note that, in a recent paper, Ramadevi et al. found that Nissle 1917 could form more the amount of biofilm compared to MG1655 at 37°C for 48 h, although two strains have a similar growth rate (Ramadevi et al., 2023). The difference may be due to the different temperatures used in our experiments. Gill et al. reported that Nissle 1917 forms biofilm in a temperature-dependent manner (Gill et al., 2019). Comprehensive analysis of the various physiological conditions, including temperature, nutrient, pH and osmotic stress, on the probiotic activity is necessary for its clinical application.

### 3.2 Biofilm morphological characterization

**Figure 2:**
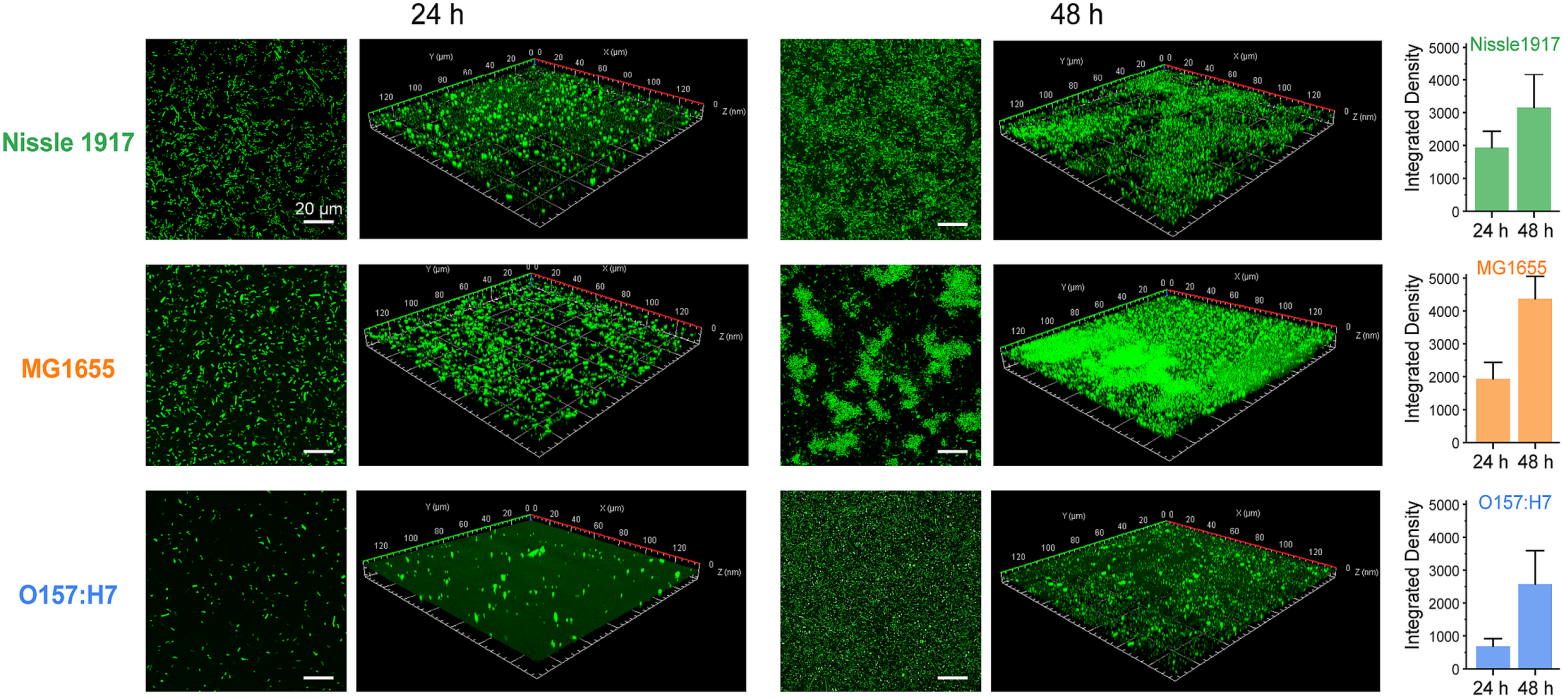
Analysis of probiotic, opportunistic and pathogenic *E. coli* biofilms using CLSM. Representative fluorescence microscopic images present the formations of biofilm by three *E. coli* strains: Nissle 1917, MG1655 and O157:H7 after 24 h and 48 h of growth, respectively. The scale bar in two-dimensional images was 20 μm; while in the three-dimensional ones, an area of 140 μm × 140 μm was examined. The integrated densities depicted on the right were calculated based on the two-dimensional images with three replicates for each treatment.

The morphologies of probiotic, opportunistic and pathogenic *E. coli* biofilms were observed using confocal laser scanning microscopy (CLSM), which has been used to map biofilms and quantify the distribution of extracellular proteins, polysaccharides, lipids, nucleic acids, and other biomolecules (Zhang et al., 2019). To visualize their growth characteristics and status, the biofilms (Nissle 1917, MG1655 and O157:H7) were allowed to grow for 24 h and 48 h, respectively, then the bacterial cells were stained with SYTO 9 and scanned under 488 nm excitation. The two-dimensional and layered three-dimensional scanning images of the three *E. coli* biofilms are shown in Figure 2. It can be seen that, at the 24 h, which corresponds to the colonization and development stage indicated in Figure 1, many bacterial cells adhere to the surface and form microcolonies in Nissle 1917 and MG1655, and the number of cells in MG1655 was higher than that in Nissle 1917, whereas only a few microcolonies adhere to the surface in O167:H7. After incubation for 48 h to the maturation stage of biofilms, Nissle 1917 colonies were connected over a large area forming thick communities of 3.53 μm; MG1655 has the thickest biofilm of 6.67 μm with many “island” shaped structures which indicating a more mature biofilm with complex three-dimensional communities; pathogenic O157:H7 has the thinnest biofilm of 1.96 μm, microcolonies were loosely dispersed. The integrated density was also calculated and shown in Figure 2 (right), it can be seen that at 24 h, the biomass of Nissle 1917 and MG1655 was greater than O157:H7; at 48 h, MG1655 had the most biofilm mass while O157:H7 had the least. These results are consistent with Figure 1, where biofilms formed by opportunistic pathogenic *E. coli* were the thickest, while pathogenic *E. coli* were the thinnest, and biofilms formed by probiotic *E. coli* were somewhere in between. It is well known that the attachment of the bacterial cell to a particular region is required for biofilm formation (Ramadevi et al., 2023). We can expect that the production of curli in probiotic *E. coli* will be modest.

**Figure 3:**
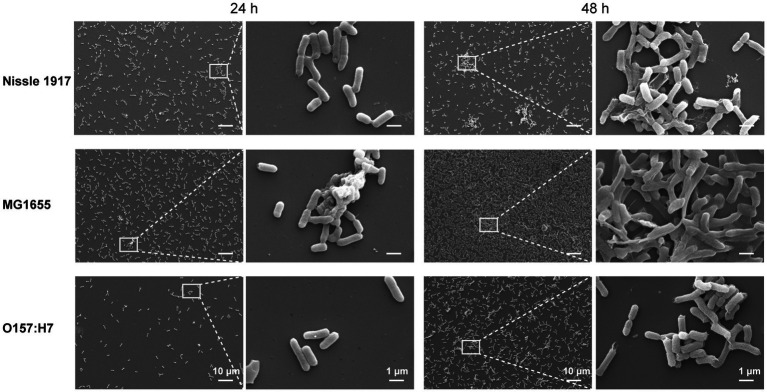
Comparison of the morphology of *E. coli* biofilms between probiotic opportunistic and pathogenic strains using scanning electron microscopy (SEM). The images depict the attachment of three *E. coli* strains, Nissle 1917 (probiotics), MG1655 (opportunistic) and OH157:H7 (pathogen), to surfaces for 24 and 48 h at 25°C.

SEM was also used to examine the differences in the spatial structure between the three *E. coli* biofilms (Figure 3). The results showed, all three bacteria adhered to the surface after 24 h. In addition, Nissle 1917 formed a single layer of microcolonies; MG1655 not only formed a few layers of microcolonies, but also started to produce extracellular polymeric substances (EPS); the number of attached microbial cells of O157:H7 is relatively less. After 48 h, the biofilms of all three bacteria were more mature: Nissle 1917 biofilms consisted of several layers of microbial cells and some EPS structures; MG1655 biofilms formed stable three-dimensional communities with channels inside; O157:H7 biofilms had fewer layers and less EPS compared to the other two. All the three *E. coli* strains formed complex bacterial communities in biofilm structures, through which they may improve their quorum sensing among microbes and increase their tolerance to survive in harsh environments (Annous et al., 2009).

### 3.3 Biofilm chemical component analysis

**Figure 4:**
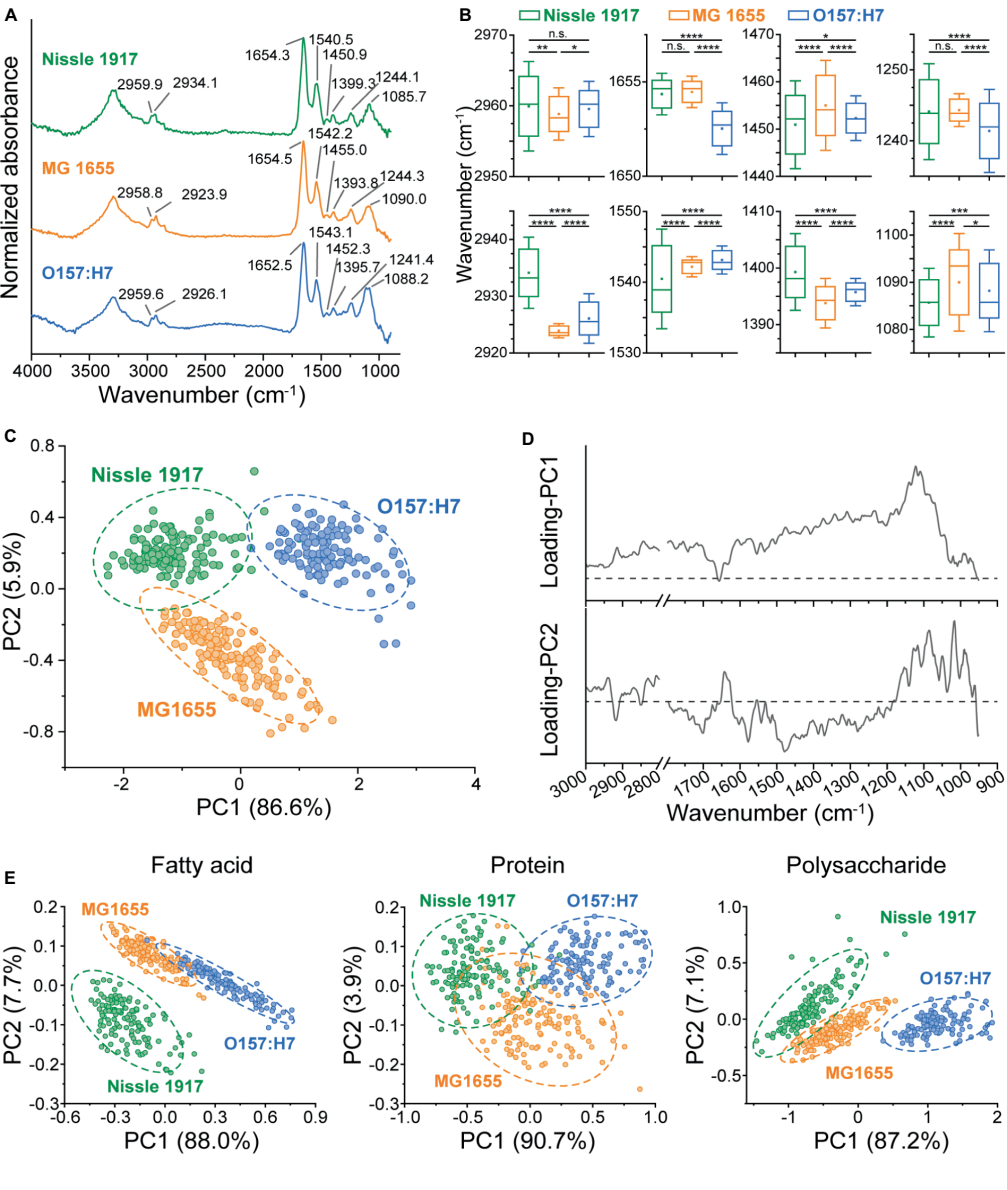
Comparison of chemical component in three *E. coli* biofilms using FTIR microspectroscopy. **(A)** The Averaged IR absorbance spectra of Nissle 1917, MG1655 and O157:H7 biofilms cultured for 24 h were labeled with the main absorption bands on the graph to show the differences in their chemical composition. **(B)** The graphs present eight absorption peak positions of the *E. coli* biofilms, with Nissle 1917, MG1655 and O157:H7 listed on the x-axis and the peak positions on the y-axis. Statistical significance is indicated as follows: ****p < 0.0001, ***p < 0.001, **p < 0.01, *p < 0.05, n.s. *p* > 0.05. A horizontal line on the boxplot represents the mean value, while error bar depicts 1.5 × SD. 140 replicates for each sample. **(C)** Principal Component Analysis (PCA) was conducted on the IR absorbance spectra of the three *E. coli* biofilms. Circles indicate the 95% confidence interval. Spectral region 3,000–2,800 cm^−1^ and 1,800–900 cm^−1^ were selected, with 100 spectra obtained per sample. The corresponding loading plots for PC1 and PC2 were presented in **(D)**. **(E)** PCA was performed on the fatty acid component (spectrum region 3,000–2,800 cm^−1^), the protein component (1800–1,500 cm^−1^) and the polysaccharide and nucleic acid component (1200–900 cm^−1^) component of the three biofilms.

The previous results indicated the differences in biomass and morphology between the three biofilms, to further explore the metabolic phenotypic differences, their chemical components were thoroughly analyzed. FTIR spectroscopy has been widely used to characterize the fatty acid, protein, polysaccharide and nucleic acid compositions in biomaterials without any complex sample preparation procedures (Cheeseman et al., 2021; Chirman and Pleshko, 2021; Sportelli et al., 2022; Wang et al., 2023b), so it was further applied to find out the biochemical molecular component differences between the three *E. coli* biofilms. MG 1655, Nissle 1917 and O157:H7 biofilms were imaged by FTIR microspectroscopy, and 140 individual IR absorption spectra of each biofilm were collected. The averaged spectra of each biofilm are shown in Figure 4A, with eight main absorption peak positions indicated on the graph. It can be seen that the absorption peaks of three biofilms were different, to find out whether the difference was statistically significant, all the 140 IR spectra of each biofilm were statistically analyzed, the results are shown in Figure 4B. Eight absorption bands were identified: 2,970–2,950 cm^−1^ (C-H asymmetric stretching vibrations of methyl groups in the fatty acids), 2,945–2,920 cm^−1^ (C-H asymmetric stretching vibrations of methylene groups in fatty acids), 1,657–1,650 cm^−1^ (amide I of α-helical structures in proteins), 1,550–1,530 cm^−1^ (amide II in proteins), 1,470–1,440 cm^−1^ (C-H deformations of methylene groups), 1,410–1,385 cm^−1^ (C=O symmetric stretching vibrations of COO^−^), 1,255–1,235 cm^−1^ (P=O asymmetric stretching vibrations of >PO_2_^−^), 1,105–1,075 cm^−1^ (P=O symmetric stretching vibrations of >PO_2_^−^) (Lasch and Naumann, 2015). The results showed that Nissle 1917 had significantly different absorption peaks with MG1655 and O157:H7 at 2934.1 cm^−1^, indicating that the methylene groups in the fatty acids of the Nissle 1917 biofilm had different components from the other two biofilms. Similarly, O157:H7 had significantly different amide I components compared to Nissle 1917 and MG1655 biofilms. So far, we do not know the biological significance of the different chemical components between these strains. However, the infrared data provide useful clues to understanding the role of chemical molecules in the biofilm formation and probiotic properties. To the best of our knowledge, this is the first report on the comparison of microbial biofilms between different strains of the same species.

In order to visualize the differences in the metabolism of the three biofilms in a more intuitive way, a PCA based on whole molecular components (3,000–2,800 cm^−1^, 1,800–900 cm^−1^) was also performed, see Figure 4C. It can be seen that the three biofilms can be completely distinguished, indicating that the biochemical molecular components are significantly different. The loading plot of the PCA was shown in Figure 4D, in principle component 1 (PC1), the bands around 1,122 cm^−1^ (corresponding to polysaccharides) contributed the most, while in PC2 the bands around 1,479 cm^−1^ (corresponding to fatty acids), 1,085 cm^−1^ (corresponding to nucleic acids) and 1,016 cm^−1^ (corresponding to polysaccharides) contributed the most. To find out which biomolecules differed between the three biofilms, PCA was performed on three spectral regions: fatty acid (3,000–2,800 cm^−1^), protein (1,800–1,500 cm^−1^) and polysaccharide & nucleic acid (1,200–900 cm^−1^), respectively (Figure 4E). It can be seen that, Nissle 1917 biofilm has significantly different fatty acid components, O157:H7 has significantly different nucleic acid and polysaccharide components, while their protein component shows partial similarities. Probiotic Nissle 1917 biofilms may secrete specific phospholipid components into the extracellular matrix, which may play an important role in establishing a healthy microenvironment in the human gut; whereas pathogenic O157:H7 biofilm may secrete specific exopolysaccharides and extracellular nucleic acids into the extracellular matrix to produce its toxicity and pathogenicity (Soliemani et al., 2022). Further analysis and identification of these intracellular or extracellular metabolites, for example by using mass spectrometry (MS), will help to elucidate the network of different metabolic pathways during biofilm formation.

**Figure 5:**
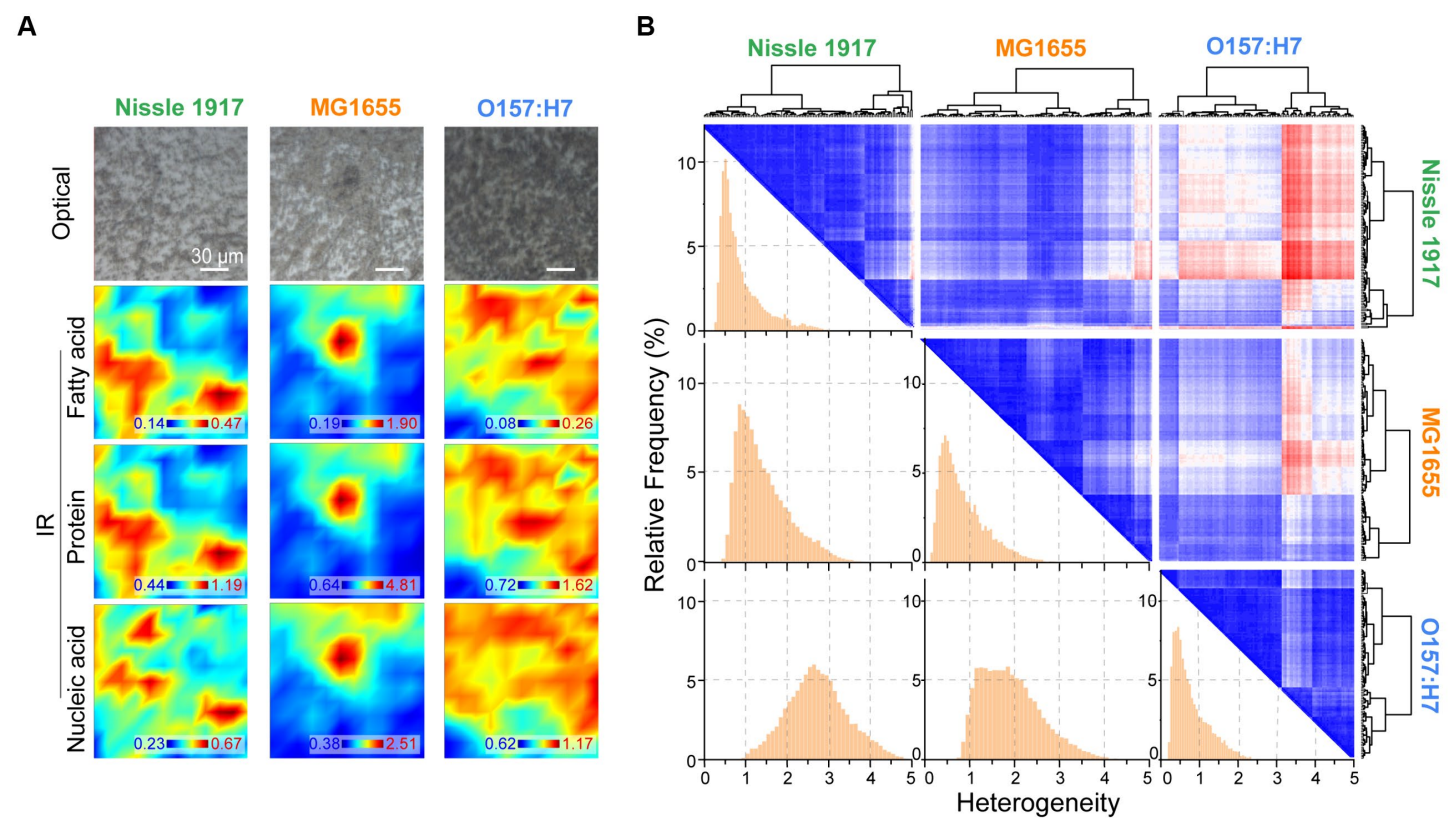
Quantitively analysis of spatial heterogeneity of three *E. coli* biofilms through FTIR microspectroscopy. **(A)** Optical and corresponding IR mapping images of Nissle 1917, MG1655 and O157:H7 biofilms were presented, IR images were detecting acquired based on the peak areas between 3,000–2,800 cm^−1^ (fatty acids), 1700–1,600 cm^−1^ (proteins) and 1,200–1,000 cm^−1^ (polysaccharides), respectively. **(B)** Heterogeneity was analyzed between each two *E. coli* biofilms based on the spatial IR absorption spectra extracted from **(A)**, three large rows and columns indicate Nissle 1917, MG1655 and O157:H7, respectively. Hierarchical cluster analysis (HCA)-heatmap was presented at the top right, and heterogeneity histograms were shown at the bottom left. The x-axis depicted the heterogeneity between two biofilms, larger values indicated higher heterogeneity.

The spatial distribution characteristics of the chemical composition of the three *E. coli* biofilms were further characterized by FTIR microspectroscopy; for each biofilm, the optical image and corresponding IR absorption images based on fatty acid, protein and nucleic acid are shown in Figure 5A. It can be seen that the spatial distribution of the biomolecules was inhomogeneous. The spatial heterogeneity of the biofilms was then evaluated quantitatively (Figure 5B). On the histogram, row 1 column 1, row 2 column 2 and row 3 column 3 indicated the within-group heterogeneity of Nissle 1917, MG1655 and O157:H7 biofilms, respectively; row 2 column 1 indicated inter-group heterogeneity of Nissle1917-MG1655, row 3 column 1 indicated Nissle1917-O157:H7, row 3 column 2 MG1655-O157:H7. The results showed that the inner-group heterogeneity of Nissle1917 and O157:H7 was relatively low, with a relatively sharp peak shape and peak positions less than 1. This proved that the spatial distribution of the biomolecules of the two biofilms was relatively uniform. The inter-group heterogeneity between Nissle1917-O157:H7 was greater than that between Nissle1917-MG1655 and MG1655-O157:H7, suggesting that the biochemical differences of biofilms between probiotic *E. coli* Nissle 1917 and pathogenic O157:H7 were much greater than those between Nissle 1917 and opportunistic MG1655. The clear biochemical heterogeneity between the three stains supports the idea that the spatial structures of the biofilms may be related to their biological activity. Further experiments are in progress.

## 4 Conclusion

In this work, we have characterized the biofilm of the probiotic strain *E. coli* Nissle 1917. Two other *E. coli* strains, pathogenic O157:H7 and opportunistic O157:H7, were used for comparison to find the unique features in the probiotic biofilm. The biofilm mass, structural features and chemical compositions of these three strains were investigated by using crystal violet assay, confocal laser scanning microscopy, scanning electron microscopy and FTIR microspectroscopy, respectively. The results showed that the probiotic strain had the better ability in biofilm formation than the pathogenic strain O157:H7, but less than the opportunistic strain MG1655. Similarly, the morphology of the probiotic biofilm formed was thicker and contained more EPS than that of the pathogenic strain. Interestingly, chemical composition analysis revealed that strain Nissle 1917 possesses the unique methylene groups in biofilm fatty acids and may secrete specific phospholipid components into the extracellular matrix. Furthermore, the biochemical heterogeneity in the probiotic Nissle 1917 biofilm is far from the pathogenic O157:H7 but close to the opportunistic MG1655. To the best of our knowledge, this is the first report on the comparison of microbial biofilms between different strains of the same species. These findings not only provide more information on the morphology, architecture and chemical composition of the probiotic biofilms, but also provide valuable clues to understanding the mechanism of probiotic action in biofilm formation for their clinical and therapeutic applications. In addition, this work also provides new insights into the role of biofilm in the beneficial or pathogenic properties of a microbe. Importantly, the application of FTIR microspectroscopy allows the spatial heterogeneity and unique chemical components in the biofilm or its matrix to be studied, providing a novel methodology for biofilm research. Given the attachment is required for biofilm formation in the human gut, the influence of host matrices on the probiotic biofilms will be an interesting topic to explore.

## Data availability statement

The raw data supporting the conclusions of this article will be made available by the authors, without undue reservation.

## Author contributions

HL: Writing – original draft, Investigation, Formal analysis. JM: Writing – original draft, Investigation. PY: Writing – original draft, Investigation. FG: Writing – original draft, Methodology. XL: Writing – original draft, Methodology. JL: Writing – review & editing, Funding acquisition, Conceptualization. YW: Writing – review & editing, Funding acquisition, Data curation, Conceptualization.
